# Evaluation of Fresh Property, Compressive Strength and Environmental Impact of Low-Carbon Geopolymer Based on Ladle Furnace Slag and Soda Residue

**DOI:** 10.3390/ma18071552

**Published:** 2025-03-29

**Authors:** Xiaoyan Liu, Yulan Zuo, Fengming Yang, Junqing Zuo, Aihua Liu, Huang Huangfu, Kai Lyu, Xian Xie, Surendra P. Shah

**Affiliations:** 1 College of Civil and Transportation Engineering, Hohai University, Nanjing 210098, China; liuaihua88@126.com (A.L.); 20200903@hhu.edu.cn (K.L.); 2 College of Material Science and Engineering, Hohai University, Changzhou 213000, China; maymayzyl@163.com; 3 Beijing Building Materials Academy of Sciences Research, Beijing 100041, China; 4 Shanghai Construction Building Materials Technology Group Co., Ltd., Shanghai 200086, China; junqingzuo@163.com; 5 Jiangsu Expressway Engineering Maintenance Technology Co., Ltd., Nanjing 223000, China; 6 Jiangsu Zhongzhi Transportation Innovation Industry Research Institute Co., Ltd., Nanjing 211500, China; huang_83626@hotmail.com; 7 School of Materials Science and Engineering, Southeast University, Nanjing 211189, China; xiexian14@163.com; 8 Center for Advanced Construction Materials, Department of Civil Engineering, University of Texas at Arlington, Arlington, TX 76019, USA; surendra.shah@uta.edu

**Keywords:** alkaline solid waste, alkali-activated, Na_2_O equivalent, embodied carbon

## Abstract

In this work, a novel method for the disposal of ladle furnace slag (LFS) and soda residue (SR) was proposed. By applying geopolymer technology, LFS and SR were used as precursors to manufacture a geopolymer with sufficient fresh and mechanical properties that can be used in construction works, such as in non-structural components like lightweight partition walls. The effects of raw material ratios and Na_2_O equivalents on the fresh properties, mechanical properties, microstructure and environmental impact of LFS-SR geopolymer (LSG) were analyzed by rheology, compressive strength, XRD, TG/DTG, SEM, and calculation of embodied carbon. The results showed that the compressive strength of LSGs increased when the SR content decreased or Na_2_O equivalent increased, and the maximum compressive strength could reach 12.0 MPa at 28 d. The hydration products of LSG were mainly C-(A)-S-H gel, C_3_AH_6_, and AFt. Notably, the C-(A)-S-H gels formed a stable cross-linked structure, and the extremely fine granular C_3_AH_6_ further filled the pores. Furthermore, AFt was generated from the interaction between LFS and CaSO_4_ rich in SR during the hydration process. The carbon calculation results indicated that the embodied carbon of LSGs was significantly lower than that of traditional cement, and the LSG containing 20% SR and 12% Na_2_O equivalent had the highest sustainability. This study proposed strategies for mitigating the environmental hazards of alkaline solid waste and improving its resource utilization, thereby promoting sustainable development in the construction industry.

## 1. Introduction

With the accelerated industrialization of society, the environmental burden caused by greenhouse gas emissions has increased significantly. Statistical data indicates that global CO_2_ emissions have reached 37.8 billion tons in 2024 [1]. The main source of carbon emissions in the cement industry occurs during the production stage; its emissions during that time account for 7–8% of global CO_2_ emissions [2]. Therefore, for several decades, researchers have been working on developing new environmentally friendly cementitious materials to substitute for traditional cement to reduce carbon emissions in the construction industry. Alkali-activated materials (AAMs, also known as geopolymers) are synthesized through the polymerization reaction that occurs between alkali solutions and precursors rich in calcium or silica-aluminum sources [3,4]. Compared to Portland cement, geopolymers are sustainable green building materials as they do not require calcination, can effectively utilize solid waste, reduce carbon emissions, and exhibit excellent mechanical strength and durability [5,6].

Ground granulated blast furnace slag [7,8], fly ash [9,10], metakaolin [11,12,13], and other aluminosilicate materials are commonly employed as raw materials in geopolymers. However, due to the scarcity of these materials and some special performance requirements, exploring new alternative precursor materials is necessary. Steel slag (SS) is a by-product of the steelmaking process, with a global production of about 169 to 254 million tons, and China accounts for approximately 50% of the global steel production; however, its utilization rate of steel slag is relatively low (only 30%) [14,15,16]. Ladle furnace slag (LFS) is a type of SS that is created during the final stage of steel production, i.e., the secondary metallurgical stage. Research shows that about 30 kg of LFS is generated for every ton of steel produced [17]. However, the predominant method of LFS treatment is simply landfilling, which has caused a huge burden on the ecosystem [18].

Considering that the primary mineral components of LFS are C_2_S and C_12_A_7_, it exhibits certain hydration activity. Meanwhile, LFS is also a high alkalinity solid waste, abundant in calcium and aluminum phases, with high hydration activity, so it presents a viable alternative to ground granulated blast furnace slag and fly ash as a new precursor material [19,20]. Therefore, some studies on LFS-based geopolymers have been carried out in recent years [21,22]. Bignozzi et al. [23] and Barbarey et al. [24] investigated the preparation of geopolymers by partially replacing metakaolin with LFS and found that the addition of LFS would improve the mechanical properties of geopolymers, achieving a maximum compressive strength of up to 21 MPa. Natal et al. [25] prepared the geopolymer by adjusting the ratio of LFS and fly ash, and its compressive strength was between 11–14 MPa. Similarly, Sing et al. [26] prepared a 10 mm thin geopolymer based on fly ash and LFS, studied its flexural properties, and found that the addition of LFS could promote the formation of C-S-H gel and improve its flexural properties. To further reduce the use of traditional precursors, Wang et al. [27] activated LFS with sodium silicate solution with a modulus of 1 and varying alkali equivalents of 4%, 6%, and 8%, and found that the compressive strength and slump flow of LFS-based geopolymers without traditional precursors improved with increasing sodium silicate content. In particular, Najm et al. [28] used the orthogonal method to investigate the changes in the setting time and compressive strength of alkali-activated LFS mortar. The addition of alkali activators shortened the setting time and increased the compressive strength, reaching 11.1 MPa. Therefore, LFS is identified as a significant solid waste material with considerable yield and certain hydration activity, and is an appropriate precursor material for geopolymers. However, previous studies mostly focused on the mechanical properties of LFS-based geopolymer, without systematically studying its setting time and rheological behavior.

Soda residue (SR) is a solid waste generated during the production of Na_2_CO_3_ via the ammonia-soda process. It is usually grayish-white, and its main chemical components include CaCO_3_, SiO_2_, Ca (OH)_2_, CaCl_2_, CaSO_4_, etc. [29]. With pH values ranging from 10 to 12, SR exhibits strong alkalinity, which is prone to leakage during storage; this risks soil salinization and water source contamination, making it a major threat to environmental integrity and human health [30]. China is the largest producer of SR in the world, with an annual output of 7.8 million tons [31]. Currently, SR is widely utilized for the recovery of heavy metals [32,33] and enhancement of the soil environment [34,35]. Therefore, the accumulation of LFS and SR faces great environmental pressure and safety hazards, and how to rationally utilize them has become an urgent issue for researchers in the field.

So far, LFS as a precursor material for geopolymers has a certain research basis for the mechanical properties, but the studies on setting time and rheological properties are still limited, and there is a lack of research on the cooperative hydration mechanism of SR and LFS. In this study, through preliminary experiments, it was determined that sodium silicate solution with the modulus (Ms) of 1.2 was used as the alkali activator to prepare low-carbon LFS and SR geopolymer material (LSG). Further, LSGs with different proportions were prepared by manipulating SR content and Na_2_O equivalent in this study, and the hydration products, rheological properties, pyrolysis characteristics, and environmental impacts were characterized by XRD, rheology, SEM, TG/DTG, and calculation of embodied carbon.

## 2. Materials and Experiments

**Figure 1:**
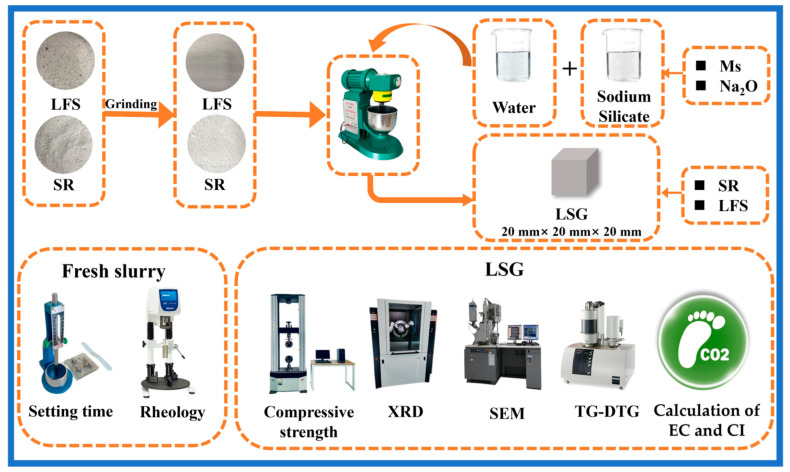
The research process of this paper.

The main processes of this research are shown in Figure 1, including specimen preparation, fresh properties testing, compressive strength test, microscopic characterization, and calculation of embodied carbon (EC) and embodied carbon index (CI).

### 2.1. Materials

**Figure 2:**
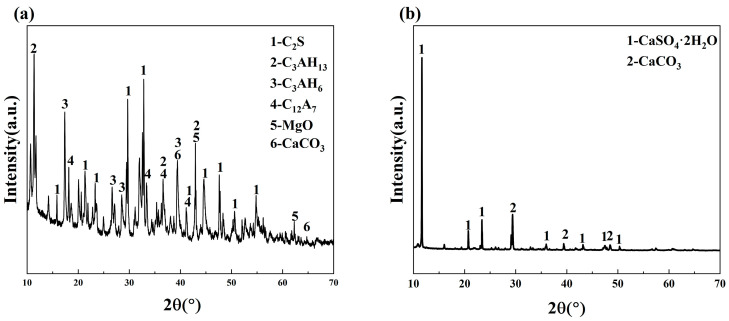
The XRD patterns of (**a**) LFS and (**b**) SR.

**Figure 3:**
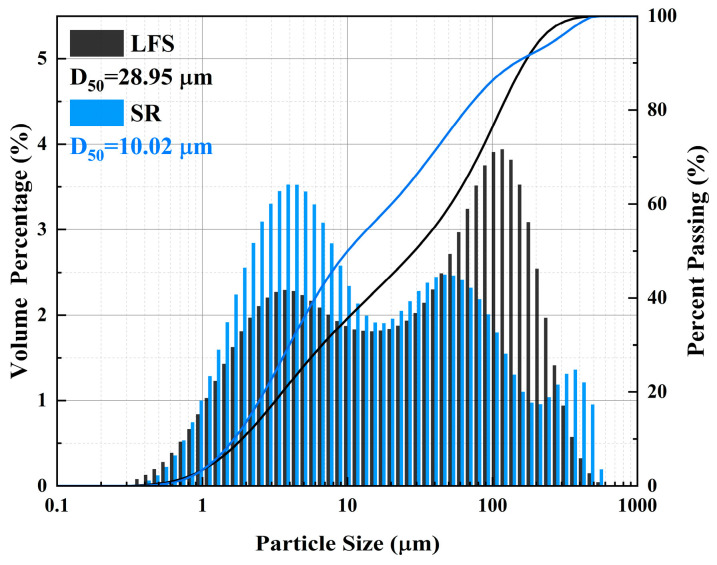
Particle size distribution of LFS and SR.

In this study, LFS was obtained from Nanjing Iron and Steel Group, China, and SR from Lianyungang, China. The chemical components of LSF and SR were determined by X-ray fluorescence spectroscopy (XRF), as shown in Table 1. The mineral composition of LFS and SR was determined by X-ray diffraction (XRD), as shown in Figure 2. CaO is the main component of LFS (51.75 wt.%) and SR (51.44 wt.%). In addition, LFS is also rich in 25.45 wt.% Al_2_O_3_ and 13.97 wt.% SiO_2_, which is suitable for using as a precursor material of geopolymers. The main mineral composition of LFS is C_2_S and C_12_A_7_, and the mineral composition of SR is CaSO_4_·2H_2_O and CaCO_3_. The particle size distribution is shown in Figure 3. D50 of LFS and SR are 28.95 μm and 10.02 μm, respectively.

The polycarboxylate superplasticizer was provided by Subote New Materials Co. Ltd., Nanjing, Jiangsu, China. The alkali activator was a sodium silicate solution. The Ms (SiO_2_/Na_2_O molar ratio) of liquid sodium silicate was 3.32. The Ms of the sodium silicate solution was adjusted to 1.2, 1.5, and 1.8 by adding a certain amount of NaOH particles (≥98% purity, analytical grade, and white particles). After the configuration, the alkali activator was cooled at room temperature for 24 h to achieve the condition of sufficient mixing and to eliminate the effect of temperature on the preparation.

**Figure 4:**
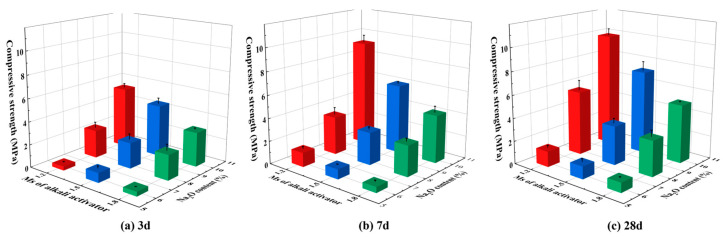
Effects of different Ms and Na_2_O equivalent of alkaline activator on the compressive strength of LSGs at 3 d, 7 d, and 28 d.

To determine the optimum parameters of the alkali activator, the sodium silicate solution with different Ms (1.2, 1.5, and 1.8) was used to activate LSG pastes (mass ratio of SR: LFS = 1:4). The water-binder ratio and superplasticizer content were kept at 0.45 and 2%, respectively, and the Na_2_O equivalent was kept at 4–10%. It is worth noting that the water in the sodium silicate solution was also considered a source of water. The compressive strength of each sample at 3 d, 7 d, and 28 d are shown in Figure 4. It can be seen that the compressive strength of the samples decreased with increasing Ms, which may be due to the decrease of NaOH concentration, resulting in a weaker alkaline environment that was unfavorable for the polymerization of the geopolymer [36]. Overall, the sodium silicate solution with Ms of 1.2 showed the best activation effect on LSG, and the compressive strength could reach 9.9 MPa at 28 d. Therefore, the sodium silicate solution with a Ms of 1.2 was selected as the alkali activator in this study.

### 2.2. Mix Proportions and Specimen Preparation

In this study, SR and LFS were used as precursors, and the SR content was 20%, 40%, and 60%, respectively, the water-binder ratio was 0.45, and the superplasticizer content was 2%. The specific mix ratio design is shown in Table 2. For example, LSG containing 20% SR and 6% Na_2_O equivalent was named A2-6. First, the alkali activator, superplasticizer and water were mixed, and then SR and LFS were mixed with liquid to prepare LSG pastes. After that, the pastes were poured into 20 mm × 20 mm × 20 mm molds and then kept at room temperature for approximately 24 h before demolding. After demolding, all samples were cured in the curing room at (20 ± 2) °C and relative humidity of (95 ± 5)% until the test age.

### 2.3. Test Methods

#### 2.3.1. Fresh Properties Test

**Figure 5:**
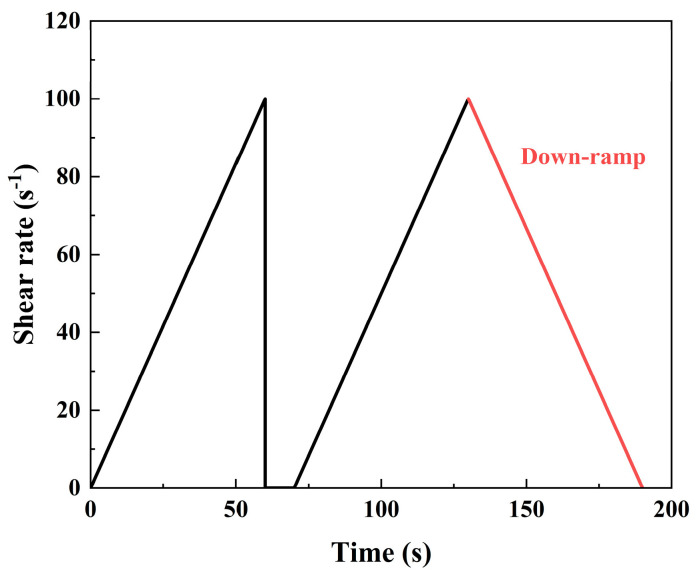
Procedure for testing rheological properties of LSG pastes [38].

The fresh properties of LSG were evaluated by setting time and rheology tests. The setting times of the fresh LSG were determined using a Vicat apparatus according to the Chinese standard GB/T 1346-2011. The dynamic yield stress and plastic viscosity of the fresh LSG were determined using a rheometer (AMETEK Brookfield, Middleborough, MA, USA). The Bingham model was used to fit the flow curve of the fresh LSG, and the rheological curve could be obtained from the shear down-ramp part [37]. The shear test was carried out using the shear scheme shown in Figure 5: the shear rate increased from 0 s^−1^ to 100 s^−1^ in the initial 60 s, and then the shear was stopped. At 70~130 s, the shear rate increased from 0 s^−1^ to 100 s^−1^, and decreased from 100 s^−1^ to 0 s^−1^ at 130 s to 190 s. The expression of the Bingham model is shown in Equation (1).

(1) $$\tau =\tau_{0}+\rho \gamma$$

where *τ* is shear stress, Pa; γ is the shear rate, s^−1^; *τ*_0_ is the yield stress obtained by fitting flow model, Pa; *ρ* is the plastic viscosity coefficients, Pa⋅s.

#### 2.3.2. Compressive Strength Test

The compressive strength of LSGs was measured at 3 d, 7 d, and 28 d, using three specimens for each group, following the testing procedure described in GB/T 17671-2021.

#### 2.3.3. Microstructure Test

After the compressive strength test of the LSGs at the age of 28 d, the broken samples were collected and soaked in anhydrous ethanol to stop hydration and then dried in the vacuum drying oven at 60 °C for 48 h. To identify the mineral compositions of the raw materials and LSG pastes, X-ray diffraction spectroscopy (Cu Kα radiation, Rigaku D/MAX-2200PC, Tokyo, Japan) was used. The scanning range was 10°~90° with a step size of 2°/min. To determine the main chemical components of LSG, the dry samples were ground, and the sample powders were analyzed by a thermogravimetric analyzer (TGA Q500, TA Instruments, New Castle, DE, USA). The temperature was increased from room temperature to 1000 °C, and the heating rate was 20 °C/min. Furthermore, LSG pieces of about 3 mm were selected and then coated with gold before the SEM test. The SEM images were obtained using a Hitachi Regulus 8100 scanning electron microscope (Hitachi High-Technologies Corporation, Tokyo, Japan).

#### 2.3.4. Calculation of Embodied Carbon

The carbon emissions of 1 m^3^ low-carbon LSG prepared by alkali activating LFS and SR were calculated by embodied carbon (EC, e-CO_2_). According to the mix ratio of this research, 1.25 t precursor materials, 0.5625 t water, and 0.025 t superplasticizer were required to prepare 1 m^3^ LSGs. It is worth noting that the EC generated by the electrical energy consumed by LFS and SR during the grinding process was also included in the total carbon emissions, and the energy consumption of grinding was 36.7 KWh/t [39]. The carbon coefficient (CC) of raw materials required for the composite paste and the conversion of electric energy are shown in Table 3. The CC values of raw materials are based on highly relevant literature to ensure their reliability. The specific calculation formula is as follows:

(2) $${EC}_{MP}=\sum_{i=1}^{n}\left(Q_{i}\times {CC}_{i}\right)$$

where *EC_MP_* is the embodied carbon per ton of raw material, kg CO_2_e/t; *i* is the raw material in the composite paste; *Q_i_* is the quality of raw material i, kg; *CC_i_* is the carbon coefficient of raw material, kg CO_2_e/t.

Furthermore, to evaluate the environmental impact of LSG, the embodied carbon index (CI) [40] was utilized to evaluate the combined impact of environmental and mechanical properties, as shown in Equation (3). The compressive strength refers to the compressive strength of samples at 28 d. In general, the pastes with a lower CI value have a lower environmental impact.

(3) $$CI=\frac{EC(\mathrm{kg}CO_{2}e/t)}{C\mathrm{ompressive}\;\mathrm{stress}(\mathrm{MPa})}$$

**Table 3:** Carbon coefficient of electricity and each raw material.

	Carbon Coefficient (CC)	Ref
LFS	63 kg CO_2_e/t	[41]
SR	7.3 kg CO_2_e/t	[42]
Water	1 kg CO_2_e/t	[41]
Sodium silicate	460 kg CO_2_e/t	[43]
Superplasticizer	720 kg CO_2_e/t	[44]
Electricity	0.7035 kg CO_2_e/kWh	[45]

## 3. Results

### 3.1. Fresh Properties

#### 3.1.1. Setting Time

**Figure 6:**
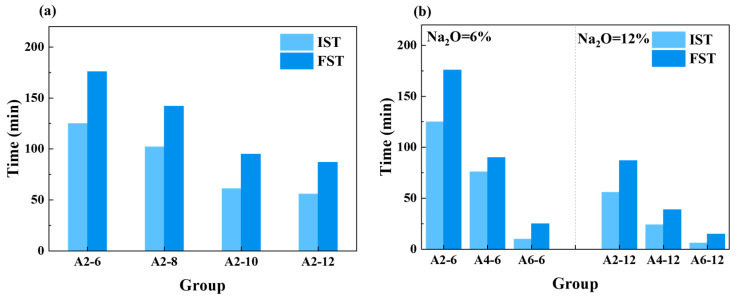
The setting time of fresh LSG: (**a**) different Na_2_O equivalent, (**b**) different SR content.

Figure 6a shows the effect of different Na_2_O equivalents on the setting time of the fresh LSG with the same SR content (20%). When the Na_2_O equivalent increased from 6% to 12%, the initial setting time (IST) of LSGs was shortened from 115 min to 56 min, which was a decrease of 52.7%, and the final setting time (FST) was shortened from 156 min to 87 min, which was a decrease of 44.2% (Figure 6a). This was due to the soluble Si in sodium silicate accelerating the dissolution of LFS and SR and speeding up the hydration rate [46].

On the other hand, the increase in SR content significantly shortened the setting time of the fresh LSG (Figure 6b). For LSGs with 12% Na_2_O equivalent, the initial setting time was shortened from 56 min to 6 min, and the final setting time was also shortened from 87 min to 15 min. This may be attributed to the high water-absorption rate of SR, and when the SR content increased, the amount of free water in the LSG with a higher SR content diminished markedly, resulting in a shorter setting time [47]. Additionally, SR was rich in CaCO_3_, which was the accelerator of the paste hydration reaction, and the increasing of Ca^2+^ accelerated the hydration of LFS and the formation of C-(A)-S-H gels in the early stage [48,49].

#### 3.1.2. Rheology Property

**Figure 7:**
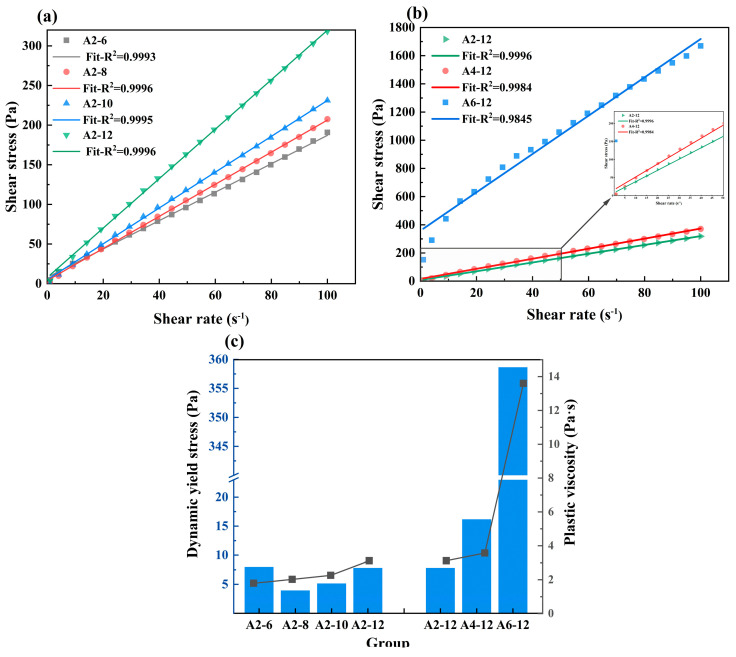
Down-ramps of shear cycles fitted with the Bingham model: (**a**) different Na_2_O equivalent; (**b**) different SR content and (**c**) the dynamic yield stress and plastic viscosity of fresh LSG.

The rheological curves of the fresh LSG were measured and Bingham model was employed to fit the down-ramp curve of the shear cycle. The rheological curves are presented in Figure 7, and the R^2^ values of all curves were between 0.9845 and 0.9996, indicating that the fresh LSG fit the Bingham model well. For the groups with 20% SR content, the fresh LSG exhibited superior fluidity (Figure 7a,c). The dynamic yield stress and plastic viscosity of the fresh LSG basically increased as the Na_2_O equivalents increased; however, this trend was not significant compared to the effect of SR content, which may be due to the formation of more hydration products from the higher concentration of the alkali activator, which resulted in a slight increase in dynamic yield stress and plastic viscosity [46,50]. With the increase in SR content, the dynamic yield stress and plastic viscosity presented a linear increase, the dynamic yield stress increased from 7.83 Pa to 358.65 Pa, and the plastic viscosity increased from 3.12 Pa·s to 13.60 Pa·s, indicating that the addition of 60% SR significantly decreased the fluidity of the fresh LSG (Figure 7b,c).

### 3.2. Compressive Strength

**Figure 8:**
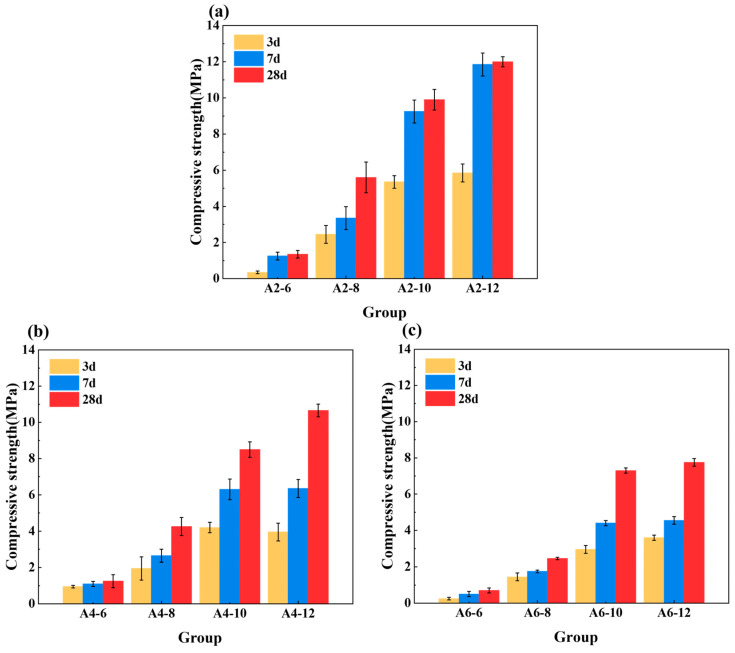
Compressive strength of LSGs at different ages: (**a**) SR content = 20%; (**b**) SR content = 40%; and (**c**) SR content = 60%.

The compressive strength of the LSGs is affected by the degree of hydration of the pastes with different proportions. Figure 8 illustrates the compressive strength results of different hardened LSGs at 3 d, 7 d, and 28 d. It was observed that the compressive strength of the hardened LSGs gradually increased as the curing age extended. Conversely, the compressive strength of the LSGs decreased when the SR content increased. Specifically, the compressive strength of the optimal groups decreased by 32.47%, 46.41%, 11.25%, 38.46%, 61.60%, and 35.42% at 3 d, 7 d, and 28 d, respectively, when the SR content was increased to 40% and 60%. LSGs with higher absorbent SR content had lower free water content, inhibiting the hydration of the pastes and reducing the compressive strength, which was also reflected in the setting time and rheological properties.

For the groups with identical SR content, the compressive strength of LSGs was improved with increased Na_2_O equivalent. The maximum strength of LSGs reached 12.0 MPa at 28 d at 12% Na_2_O equivalent. This improvement was attributed to the accelerated dissolution rate of Si/Al oxides in the high alkalinity system, which enhanced the hydration reaction, and thus, significantly improved the early strength of the geopolymer [51]. Therefore, the optimal proportions of SR and Na_2_O equivalent were 20% and 12% in the alkali-activated SR and LFS system.

### 3.3. Microstructure Analysis

#### 3.3.1. XRD Analysis

**Figure 9:**
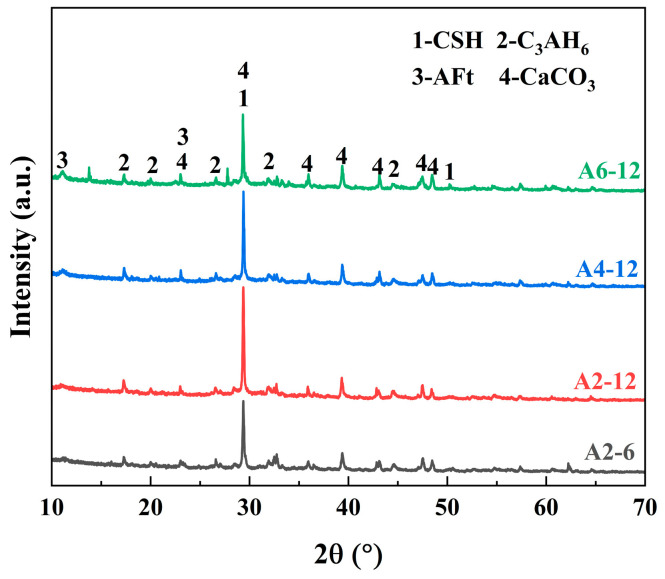
XRD results of LSGs at 28 d.

The main hydration products of LSGs with different mix ratios at 28 d were determined by XRD. As shown in Figure 9, the main mineral phase diffraction peaks of LSGs included C-S-H, C_3_AH_6_, AFt, and CaCO_3_. C_3_AH_6_ was a stable hydration product, generated from the hydration of C_12_A_7_ in LFS to the further transformation of unstable C_2_AH_8_ [52]. It is worth noting that this transformation of the crystal structure also contributed to a denser paste structure and provided early strength for LSG, according to previous research [19]. Furthermore, LFS reacted with CaSO_4_ in SR to form AFt during hydration, so the characteristic peak of AFt can be observed in the XRD pattern [53], and along with the higher SR content, the characteristic peaks of AFt were more obvious. In addition, CaCO_3_ was mainly derived from unreacted inert calcite in raw materials and carbonization of hydration products. For the groups with lower SR content and higher Na_2_O equivalent, the diffraction peaks of C-S-H were more pronounced, aligning with the compressive strength test results in Section 3.2.

#### 3.3.2. TG/DTG Analysis

**Figure 10:**
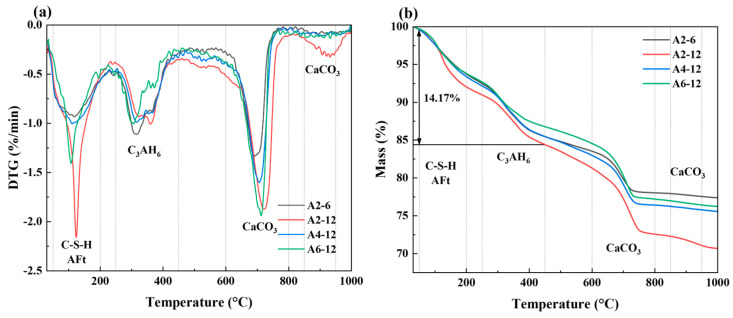
DTG (**a**) and TG (**b**) curves of LSGs at 28 d.

The TG curves represent the weight loss of LSGs during the heating process, and the DTG curves represent the weight loss rate of samples at different temperatures. The TG and DTG curves of alkali-activated LSGs at 28 d are shown in Figure 10, including three obvious weight loss peaks and a minor weight loss peak. The endothermic peaks at 50–200 °C are mainly caused by the dehydration of C-(A)-S-H gel [54,55], the endothermic peaks at about 320 °C correspond to the decomposition of hydration product C_3_AH_6_ [56], and the endothermic peaks at 600–800 °C and 850–950 °C are attributed to the decomposition of calcite [54]. The total mass loss of hardened LSGs increased with decreasing in SR content and increasing in Na_2_O equivalents. The total mass loss of group A2-12 was the highest at 29.35%, and the total content of the hydration products AFt, C_3_AH_6_, and C-(A)-S-H gels was the highest (14.17%), indicating that the LSGs in group A2-12 had the highest hydration degree and the most compact internal structure, which was consistent with the compressive strength test results in Section 3.2.

#### 3.3.3. SEM Analysis

**Figure 11:**
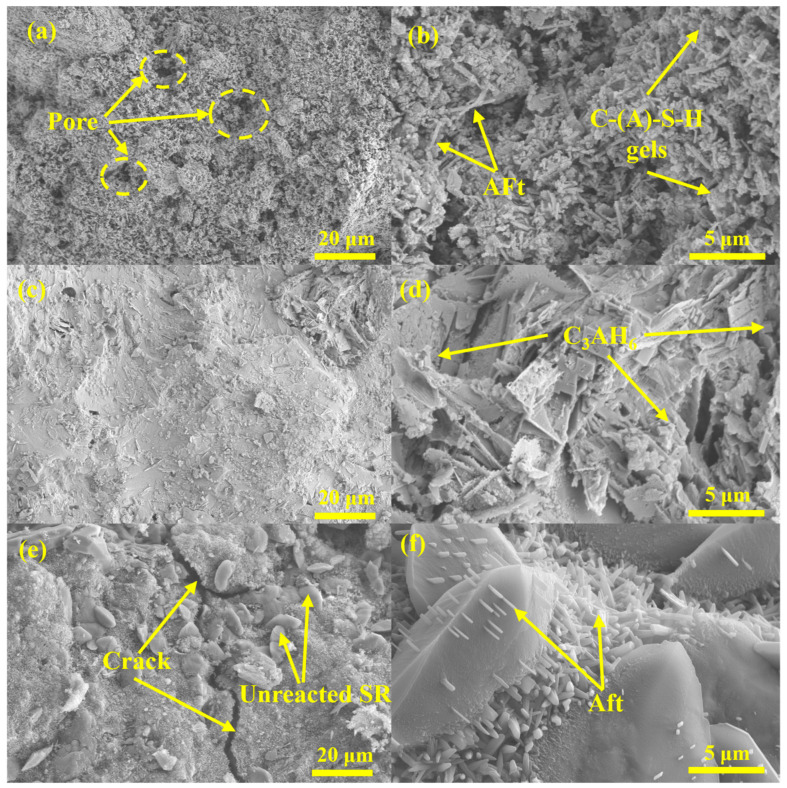
SEM image of LSG samples at 28 d: (**a**,**b**) A2-6; (**c**,**d**) A2-12; (**e**,**f**) A6-12.

The microstructure morphology of A2-6, A2-12, and A6-12 samples after curing for 28 d was detected by SEM, and the results are shown in Figure 11. When the alkali activator is added to the system, the glass phase in the raw material is destroyed, leading to the rapid release of Ca, Si, and Al and the formation of C-(A)-S-H gels, which is the main factor affecting the development of mechanical strength of each sample. In addition, it can be observed from Figure 11a,b that the A2-6 sample exhibited larger pores and loose accumulation of AFts and C-(A)-S-H gels, which may be attributed to the incomplete dissolution of Ca, Si, and Al oxides in the system at low concentrations of the alkali activator, resulting in less formation of hydration products, lower degree of polymerization, and looser microstructures. The increase in the alkali activator content facilitated the formation of the geopolymer network. Consequently, when the Na_2_O equivalent reached 12% (Figure 11c,d), the group A2-12 with the densest structure generated the largest amount of C-(A)-S-H gels and only a small number of micropores, which was consistent with the compressive strength test results in Section 3.2. Simultaneously, tiny cube-like crystals of C_3_AH_6_ were observed in the A2-12 group, filling the pores, which was in agreement with previous research [57]. On the other hand, when SR content reached 60%, wide cracks and a large number of unreacted SR particles were observed in the A6-12 sample (Figure 11e,f), resulting in a less compact structure and a subsequent deterioration in mechanical properties. In addition, more loose prismatic AFts were attached to the surface of unreacted SR particles in the A6-12 sample, destroying the dense structure of samples and leading to the strength loss of LSGs [58].

### 3.4. Calculation of Embodied Carbon

**Figure 12:**
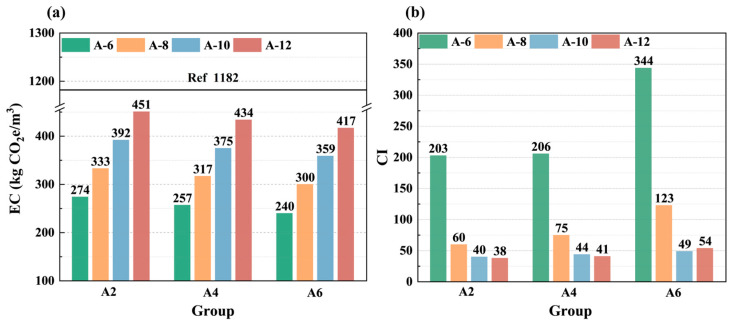
EC value (**a**) and CI value (**b**) of LSGs.

As described in Section 2.3.4, EC and CI were used to evaluate the environmental impact and sustainability of LSGs. The results of the EC calculations for LSGs were presented in Figure 12a, where the black dashed line indicated the EC value (1182 kg CO_2_e/m^3^) of the pure cement paste in the same case. The incorporation of SR reduced the EC of LSGs. The higher EC values for the high Na_2_O-equivalent group compared to the low alkali equivalent group were attributed to the higher carbon emissions of sodium silicate relative to the solid waste raw materials. That is to say, the increase of Na_2_O equivalent caused additional carbon emissions but favored the development of strength properties of LSGs. As illustrated in Figure 12b, the CI values of LSGs with different SR content exhibited a gradual increase with alkali equivalent. Notably, the CI value of group A2-12 was the lowest, indicating its superior sustainability. Furthermore, the EC values of all groups remained below 460 kg CO_2_e/m^3^, representing a 62% reduction compared to the EC value of the pure cement paste under the same conditions. Overall, LSG is a kind of low-carbon geopolymer material with high sustainability.

## 4. Discussion

**Figure 13:**
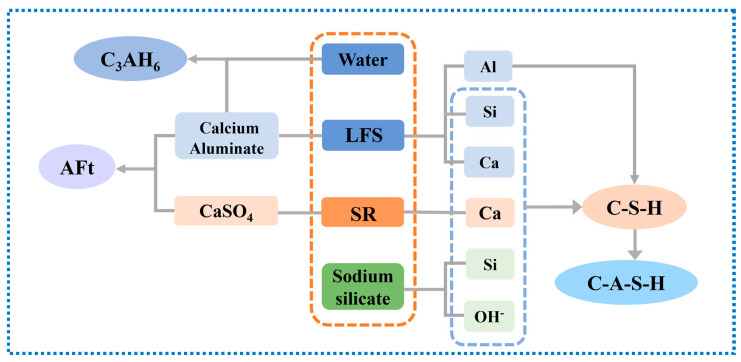
Hydration reaction mechanism of LSG.

Compared to previous studies on alkali-activated materials [23,24], our work used LFS and SR to prepare fully solid waste-based geopolymer, eliminating the need for traditional precursor materials. This study emphasizes the complementary properties of LFS and SR, providing a more comprehensive understanding of their combined utilization (Figure 13). The LSG system benefited from the abundant Al source in LFS, which compensated for the deficiency of an Al source in SR. Additionally, the incorporation of sodium silicate solution provided a stable alkaline environment for the initial reaction and an abundant Si source for the precursor, consistent with the findings of Adesanya et al. [21]. Due to the poor thermodynamic stability and larger internal specific surface area of Ca-O bonds, these bonds preferentially break over Si-O and Al-O bonds to form C-(A)-S-H gels [38]. Meanwhile, the hydration of calcium aluminate in LFS generated granular C_3_AH_6_. Furthermore, CaSO_4_ in SR reacted with calcium aluminate in LFS to form AFt. The prismatic AFt acts as a skeletal framework within the LSG, while granular C_3_AH_6_ fills the pores in the C-(A)-S-H gels, collectively contributing to the dense microstructure of LSG. Therefore, the maximum compressive strength of LFS at 28 days can reach 12 MPa, which means that LSG can be used in some low-strength components, such as lightweight partition walls. In other research, the SR and Furnace slag geopolymer prepared in before research only 8.84 MPa [59].

However, excessive AFts may adhere to the surface of the unreacted precursor particles, thus destroying the dense structure of LSG, as also observed in the study of An [58]. Moreover, the high water-absorption of SR may adversely affect the fresh properties of LSG, resulting in the insufficient dissolution of the precursor and hindering the further hydration process of LSG, consistent with the research of Xu [47]. The optimal LSG formulation, with 20% SR and 12% Na_2_O equivalent, demonstrated excellent synergistic activation effects.

In the subsequent research, the water-to-binder ratio or the proportion of water-reducing agents could be appropriately increased to improve the workability of LSG and further increase the maximum dosage of SR to further reduce the environmental impact of LSG.

## 5. Conclusions

To exploit the potential of resource utilization of alkaline solid waste LFS and SR and to improve the sustainability of the construction industry, the present work adopted sodium silicate solution by alkali activation for the preparation of low carbon geopolymer materials. The effects of SR content and alkali equivalent on the fresh properties and compressive strength of LSG were systematically investigated. Furthermore, the hydration products and microstructure of LSG were analyzed by microstructural test to elucidate the synergistic hydration mechanism between LFS and SR. Additionally, the environmental impact of LSG was evaluated by comparing its EC and CI with traditional cement. The main conclusions are as follows:

(1) With the increase of Na_2_O equivalent and SR content, the setting time of fresh LSG was shortened, and the rheological properties decreased. When the SR content reached 60%, the fresh LSG exhibited rapid setting and a significant decrease in fluidity. However, the fresh LSG containing 20% SR demonstrated preferable fluidity, which was suitable for further hydration reaction and sample molding of LSG.

(2) The compressive strength of LSGs decreased as the SR dosage increased, and it increased when the Na_2_O equivalent increased. Group A2-12 displayed the best mechanical property, with the largest 28 d compressive strength of 12.0 MPa.

(3) The hydration products of LSG were mainly C-(A)-S-H gel, C_3_AH_6_, AFt, and CaCO_3_. LFS provided reactive aluminosilicate phases, while SR contributed Ca^2+^ and SO_4_^2−^ ions, promoting the formation of C_3_AH_6_, Aft, and C-(A)-S-H gel. However, superabundant SR led to the excessive generation of AFt, which damaged the dense structure of the LSGs. More hydration product formation and a denser cross-linked structure were observed in samples with low SR content and high Na_2_O equivalent, and the granular C_3_AH_6_ can further fill the pores, contributing to a denser microstructure and superior mechanical properties.

(4) The addition of SR reduced the total EC of LSGs, and the EC of each group was kept below 460 kg CO_2_e/m^3^, which was significantly lower than that of the pure cement paste under the same conditions, with the lowest CI value and the highest sustainability in the A2-12 group.

In general, alkali-activated geopolymer materials with certain fresh and mechanical properties can be prepared effectively by modulating the proportions of LFS, SR, and alkali activator. It indicated the application potential of alkaline solid wastes LFS and SR as low-carbon geopolymer materials in the construction industry, while also offering recommendations for their resource utilization.

## Figures and Tables

**Table 1:** Chemical composition of LFS and SR (wt.%).

	CaO	Al_2_O_3_	SiO_2_	MgO	SO_3_	Fe_2_O_3_	TiO_2_	MnO	Cl	Na_2_O	P_2_O_5_	LOI
LFS	51.75	25.45	13.97	3.75	2.35	1.20	0.73	0.29	0.18	0.12	0.05	0.16
SR	51.44	4.79	11.79	12.49	11.76	2.95	0.28	0.26	3.19	0.49	0.14	0.42

**Table 2:** Experimental mix design of LSG pastes.

Sample	Ms of Alkali Activator	Na_2_O (%)	SR (%)	LFS (%)	Water-to-Binder	Superplasticizer (%)
A2-6	1.2	6	20	80	0.45	2
A2-8	1.2	8	20	80	0.45	2
A2-10	1.2	10	20	80	0.45	2
A2-12	1.2	12	20	80	0.45	2
A4-6	1.2	6	40	60	0.45	2
A4-8	1.2	8	40	60	0.45	2
A4-10	1.2	10	40	60	0.45	2
A4-12	1.2	12	40	60	0.45	2
A6-6	1.2	6	60	40	0.45	2
A6-8	1.2	8	60	40	0.45	2
A6-10	1.2	10	60	40	0.45	2
A6-12	1.2	12	60	40	0.45	2

## Data Availability

The original contributions presented in this study are included in the article. Further inquiries can be directed to the corresponding author.
